# The collective impact of rare diseases in Western Australia: an estimate using a population-based cohort

**DOI:** 10.1038/gim.2016.143

**Published:** 2016-09-22

**Authors:** Caroline E. Walker, Trinity Mahede, Geoff Davis, Laura J. Miller, Jennifer Girschik, Kate Brameld, Wenxing Sun, Ana Rath, Ségolène Aymé, Stephen R. Zubrick, Gareth S. Baynam, Caron Molster, Hugh J.S. Dawkins, Tarun S. Weeramanthri

**Affiliations:** 1 Office of Population Health Genomics, Public Health Division, Department of Health, Government of Western Australia, Perth, Australia; 2 Data Linkage Branch, Purchasing and System Performance, Department of Health, Government of Western Australia, Perth, Australia; 3 Epidemiology Branch, Public Health Division, Department of Health, Government of Western Australia, Perth, Australia; 4 Centre for Population Health Research, Curtin University, Perth, Australia; 5 INSERM, US14, Paris, France; 6 Faculty of Education, University of Western Australia, Perth, Australia; 7 Telethon Kids Institute, Perth, Australia; 8 Genetic Services WA, King Edward Memorial Hospital, Perth, Australia; 9 School of Paediatrics and Child Health, University of Western Australia, Perth, Australia; 10 Western Australian Register of Developmental Anomalies, King Edward Memorial Hospital, Perth, Australia; 11 Institute of Immunology and Infectious Diseases, Murdoch University, Perth, Australia; 12 School of Pathology and Laboratory Medicine, University of Western Australia, Perth, Australia; 13 Centre for Comparative Genomics, Murdoch University, Perth, Australia; 14 Public Health Division, Department of Health, Government of Western Australia, Perth, Australia

**Keywords:** cost, data linkage, epidemiology, hospitalizations, rare disease

## Abstract

**Purpose:**

It has been argued that rare diseases should be recognized as a public health priority. However, there is a shortage of epidemiological data describing the true burden of rare diseases. This study investigated hospital service use to provide a better understanding of the collective health and economic impacts of rare diseases.

**Methods:**

Novel methodology was developed using a carefully constructed set of diagnostic codes, a selection of rare disease cohorts from hospital administrative data, and advanced data-linkage technologies. Outcomes included health-service use and hospital admission costs.

**Results:**

In 2010, cohort members who were alive represented approximately 2.0% of the Western Australian population. The cohort accounted for 4.6% of people discharged from hospital and 9.9% of hospital discharges, and it had a greater average length of stay than the general population. The total cost of hospital discharges for the cohort represented 10.5% of 2010 state inpatient hospital costs.

**Conclusions:**

This population-based cohort study provides strong new evidence of a marked disparity between the proportion of the population with rare diseases and their combined health-system costs. The methodology will inform future rare-disease studies, and the evidence will guide government strategies for managing the service needs of people living with rare diseases.

*Genet Med* advance online publication 22 September 2016

## Introduction

Rare diseases (RD) are a disparate group of disorders that can affect any body system. Most RD have a genetic association and are often severely debilitating; they impair physical and mental abilities and shorten life expectancy. As such, RD pose a challenge for the families living with these conditions and the medical community.^1^

It has been argued that RD should be recognized as a public health priority.^2,3^ It is commonly quoted that, combined, RD affect 6–8% of the population; however, there are limited data supporting this figure.^4^ Furthermore, the true burden of RD is difficult to estimate. This has resulted in a lack of recognition of the importance of RD for health-care planning and resource allocation.^5^ To date, only a few population-level studies of the impact of RD have been published. One such recent study of a population-based registry in Italy found that RD are responsible for nearly twice as many years of life lost in the population as diabetes.^6^

Epidemiological information regarding RD is challenging to collect for a number of reasons. Several thousands of RD are described, but the exact figure is impossible to establish because it depends directly on what is defined as an RD. Several definitions of RD are in use around the world, usually with a threshold of rarity based on either a prevalence or a maximum number of patients in a region.^7,8,9^

One cost-effective approach to collecting information regarding RD would be to use longitudinal population or health administrative data sets.^6,10,11^ However, routine data-collection systems are not without limitations for identifying people with RD. The most significant issue concerns the coding and classification of RD.^5^ The International Classification of Diseases (ICD) provides specific coding for only a limited number (approximately 5.1%) of RD.^5^ In the 10th revision of the ICD, one code may be shared by multiple rare and non-RD, or a single disease may require a specific combination of codes to be accurately identified. Furthermore, there are issues regarding physician knowledge and use of appropriate codes for certain RD.^5,12^ These issues affect the accuracy and comprehensive recording of RD and hamper data ascertainment.

To address this issue, the international consortium Orphanet has developed a comprehensive classification and coding system for RD called Orpha codes.^13^ Orpha codes identify clinically unique and distinct entities with prevalence equal to no more than 1 in 2,000 in the general European population. The advantages of the system have been recognized to the extent that they now form the basis for the classification of RD in the next release of the ICD (ICD-11).^13^ However, the use of Orpha codes is limited by the fact that most routine data-collection systems do not use these codes as a classification tool. Orphanet has cross-referenced Orpha codes to ICD-10, which has helped to address this limitation, but use of the Orphanet classification with systems using ICD-10 is still limited. In Australia, beginning 1 July 1999, routine data-collection systems have been using an Australian modification of ICD-10 called ICD-10-AM, which has not been cross-referenced to Orpha codes.

In Western Australia (WA), the Department of Health maintains numerous administrative data collections from services such as hospitals, health services, and practitioners. These routine data sets, in combination with data-linkage infrastructure,^14^ mean that WA has a unique capacity to identify and follow up with people living with RD and to address some of the gaps in epidemiological data regarding the collective impact of RD.

The aim of the WA RD study was to develop a resource set based on Orpha codes that could be used to investigate RD in WA. The resource set was used to interrogate the WA Hospital Morbidity Data Collection (HMDC) to identify a cohort of people in order to investigate (i) the types of RD reported, (ii) the numbers of patients with RD in WA, (iii) utilization of particular health services, and (iv) the cost of RD to the WA health system. This paper provides an overview of the study methodology, describes cohort demographics, and provides the first snapshot of health-service utilization of a cohort of people with RD.

## Materials and Methods

### RD resource set

As a result of the limitations of using Orpha codes in the Australian context, it was necessary to undertake work to first match Orpha/ICD-10 codes to ICD-10-AM codes and validate the matching process. We have named the outcome of this work the RD resource set.

Because the cohort for the study was to be selected from the WA HMDC,^15^ we used information from this data collection to assist with the creation of the RD resource set. When developing the resource set, an RD was defined as any noninfectious disease with a prevalence of less than 1 in 2,000 in the general WA population.

We started with 585 RD Orpha codes identified by Orphanet and specifically mapped to one or a set of ICD-10 codes. With guidance from clinical coders, these ICD-10 codes were matched to their equivalent ICD-10-AM codes. A process of “back translation” to Orpha codes was then undertaken to ensure that the ICD-10-AM codes described the same diseases as those captured by the Orpha codes. In some cases, because of discrepancies between the ICD-10 and ICD-10-AM coding systems, the ICD-10-AM code(s) that specifically corresponded to an Orpha code was different from the ICD-10 code provided by Orphanet. Decisions regarding how to assign ICD-10-AM codes for these diseases were made in conjunction with WA medical coding experts.

Each Orpha code was classified into a disease category or medical specialty. Orphanet classifies entities following a principle of polyhierarchy; a disorder is assigned to all categories corresponding to the medical specialties to which it is relevant. However, to avoid multiple counting during data analysis, a set of formalized rules provided by Orphanet^16^ was used to linearize the classifications such that each Orpha code was assigned to one of the 22 Orphanet classifications.

We then undertook the process of reviewing the RD resource set in keeping with the definition of an RD used in this study. First, a total of 96 Orpha codes that were assigned to the infectious-disease classification were removed from the resource set. Second, to overcome potential differences between the European and WA populations, we estimated the period prevalence for each RD in the resource set during the study period. For these prevalence calculations, data regarding the number of people who were discharged from hospital for each RD from 1 July 1999 to 31 December 2010 were extracted from HMDC and divided by the WA population size at the midpoint of the period (March 2005). For diseases with a period prevalence higher than 1 in 2,000, clinical guidance was sought regarding whether each disease should be included in the resource set used for cohort selection. A total of 22 diseases were determined to be not rare in WA and were therefore removed from the resource set.

The final RD resource set contained 467 Orpha codes and their assigned Orphanet classification, cross-referenced to 1,084 ICD-10-AM codes. The resource set is provided in the **Supplementary Materials and Methods** online.

### Study population and data sources

This retrospective cohort study included all individuals who had an HMDC record with one of the 1,084 ICD-10-AM codes from the RD resource set recorded in any diagnosis field and a discharge date between 1 July 1999 and 31 December 2010.

All inpatient hospital records for each member of the cohort for the same time period were also extracted. Variables extracted included, but were not limited to, month and year of birth, sex, all diagnosis fields, admission and discharge dates, and postal code. The Western Australian Registry of Births, Deaths, and Marriages was used to identify those in the cohort who had died during the study period.

These data were linked via probabilistic linkage using common identifiers, including name, address, and birth date.^14^ Multiple linkage passes were conducted to minimize both false-positive and false-negative errors, followed by a clerical review to resolve doubtful links. The procedures used for the extraction of data from the WA Data Linkage System have been internationally accepted as best practice.^15^

### Data analysis

To facilitate international comparisons, RD were analyzed by Orpha codes. Statistics describing the frequency and characteristics of patients, discharges, and RD diagnostic categories were calculated for the entire 11.5-year study period. Descriptive statistics for length of stay and costs of discharges were calculated. Because of the skewed nature of length-of-stay data, both mean and medians are presented.

The age for each patient was calculated on either 31 December 2010 or date of death, whichever was earlier. The following age groups were used: younger than 1 year, 1–4 years, and then subsequent 10-year age groups until >85 years. Geographical location was categorized as either metropolitan or “rural and remote” and was determined by a person's last known postal code of residence.

Hospital-discharge information is presented for all discharges and for the subgroup of discharges that had RD codes recorded in any diagnosis field (RD-related hospital discharges). This was done to capture the full picture of inpatient hospitalization activity for people with RD, in light of the well-documented issues with accurate coding of RD (see Introduction). Hospital discharges for the cohort were examined for the entire study period (1 July 1999 to 31 December 2010). Additionally, a “2010 cohort” was assembled from a 1-year data set of people discharged between 1 January 2010 and 31 December 2010. Compilation of this 2010 cohort enabled population data comparisons to all discharges in WA during that year and facilitated cost calculations.

Costs in Australian dollars (AUD) were calculated by applying average cost weights^17,18^ to the Australian Refined Diagnosis-Related Group (AR-DRG) for each discharge during 2010 (each hospital discharge is assigned an AR-DRG, a classification system used to group patients with similar clinical characteristics requiring comparable hospital services). Using these costs, the total cost of all hospital discharges and the total cost of all RD-related hospital discharges for 2010 were calculated. These costs were compared with those of all hospital discharges in WA during the same year. Analysis for this study was conducted using SAS version 9.3 (SAS Institute, Cary, NC).

### Ethics

The study was approved by the Human Research Ethics Committee of the WA Department of Health (approval 2012/74).

## Results

### Whole cohort description

The whole cohort consisted of 61,279 people discharged from a hospital in WA with at least one RD recorded in the diagnosis fields of their hospital record during the period 1 July 1999 to 31 December 2010. There were slightly more males (52.5%) than females (47.5%) in the whole cohort, and the mean and median ages were 50.5 years (SD, 27.4) and 55 years, respectively. The majority (99%) had a WA residential postal code recorded. Of the patients with a recorded WA residential postal code, 78.4% resided in the metropolitan area and 21.6% resided in rural and remote areas. This mirrors the geographical distribution of the WA general population.^19^

Approximately 94% (441) of the 467 Orpha codes in the RD resource set were identified in the whole cohort during the study period. The majority of the cohort (87.1%) had only one RD code recorded anywhere in their HMDC records; the remaining 12.9% had 2–12 RD codes recorded. The most common Orphanet classification was rare developmental defects during embryogenesis (19.1%), followed by rare neoplastic disease (14.5%) and rare neurological diseases (12.4%). **Table 1** displays the demographic profile of those in each Orphanet classification. The table shows that a person identified as having more than one RD code could be assigned more than one Orphanet classification. However, if a person had more than one RD code in an Orphanet classification, they were counted only once in that classification.

**Figure 1:**
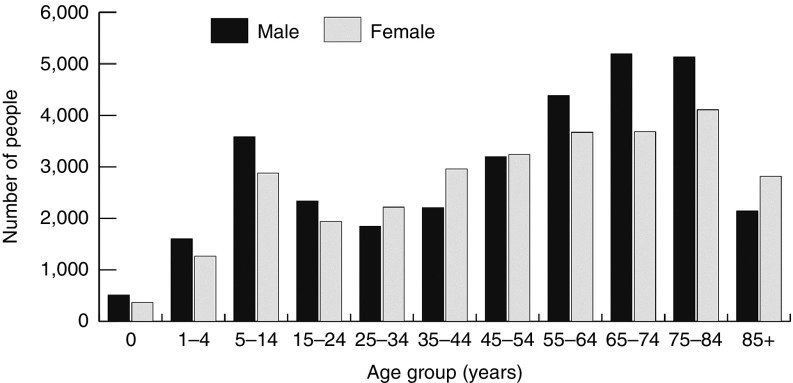
**Age and gender distribution of the whole cohort on 31 December 2010.** The demographic distribution of members of the whole cohort who were alive on 31 December 2010 (*n* = 45,213). This group represents approximately 2.0% of the WA population during 2010.

A total of 16,066 deaths were recorded among cohort members over the study period; 45,213 members were alive (73.8%) as of 31 December 2010. The WA population for the same time point was 2,294,411.^20^ Consequently, in December 2010 the whole cohort represented 2.0% of the WA population. The age and gender distributions of members alive as of 31 December 2010 are displayed in **Figure 1**.

### The whole cohort: hospital inpatient service use

The 61,279 cohort members accounted for a total of 912,492 hospital discharges, of which just over one-quarter (26.5%, *n* = 242,099) were RD-related (i.e., had at least one RD code in a diagnosis field for that discharge) during the 11.5-year study period. The mean length of stay for all cohort discharges was 3.8 days; for the subset of RD-related discharges, it was 5.5 days.

### The 2010 cohort: hospital inpatient service use and costs

Approximately one-third (20,946) of the patients in the whole cohort were discharged from hospital during 2010, accounting for 88,515 discharges (“2010 cohort” in **Table 2**). The 2010 cohort represented 4.6% of the total number of patients discharged form hospital and 9.9% of all discharges in WA during that period. The mean number of discharges per patient during 2010 for the 2010 cohort was twice that of all people discharged from hospital in WA. For RD-related discharges only, the mean number of discharges per patient was only slightly more than that of all people discharged from hospital in WA. The mean length of stay for all discharges during 2010 for the 2010 cohort was 3.6 days; for the subset of RD-related discharges, it was 5.5 days. For comparison, the mean length of stay for all hospital discharges in WA in 2010 was 2.9 days.

Rare neoplastic diseases contributed the most to the number of RD-related discharges for the 2010 cohort during 2010 (**Table 3**). There were almost four times as many discharges coded with rare neoplastic diseases than the three next highest classifications (rare hematologic, rare developmental defect during embryogenesis, and rare neurologic diseases).

The longest mean length of stay for RD-related discharges for the 2010 cohort was recorded for rare endocrine diseases (33.5 days), although the median length of stay for this classification was substantially shorter (1 day). Rare respiratory, hepatic, neurologic, and cardiac diseases and rare developmental defects during embryogenesis were the only classifications with a median length of stay longer than 1 day.

By applying the cost weights to the AR-DRGs, the total cost of all hospital discharges and the total cost of all RD-related hospital discharges for the 2010 cohort were calculated. The total cost associated with hospital discharges for the 2010 cohort during 2010 was AUD 394,947,610, which was 10.5% of the total WA inpatient hospital expenditure (AUD 3,759,339,298) (**Table 2**). Analysis of the subset of RD-related hospital discharges in 2010 showed that the cost was AUD 173,322,256, which was 4.6% of the total WA inpatient hospital expenditure. Interestingly, the cost per discharge for RD-related discharges was approximately AUD 3,000 higher than the cost per discharge for all WA discharges.

## Discussion

This study identified a group of RD patients from an administrative data set and extracted valuable linked information about their health-service usage. The figures reported here provide striking new evidence of the collective impact of RD on the state's health system and the patients it serves. Our findings of a marked disparity between the proportion of the population with an RD and their combined cost to the inpatient hospital system will raise awareness among medical professionals and broader health departments regarding RD. Moreover, the study methodology is generalizable to other populations and could support further international epidemiological studies of the collective impact of RD.

The whole cohort in this study represents approximately 2.0% of the state population in 2010; however, they had a higher mean number of hospital discharges, a longer mean length of stay than the general population, and account for between 4.6 and 10.5% of state inpatient hospital costs for that year. This disparity not only contributes to the immediate cost of hospital care but also will have consequential social and economic implications for patients, carers, and family members. For reasons outlined here, our study cohort is probably an underestimate of the number of people with an RD, and the total cost of inpatient hospital service use by this group would be noticeably greater than that reported here. Furthermore, with the inclusion of outpatient, general practitioner, mental health, emergency, and allied health-service costs, the total health-system cost attributed to RD is expected to be substantially greater.

Data from this study can be broadly extrapolated to demonstrate the potential implications of RD for the whole of Australian inpatient hospital discharges. In 2010, the WA population was approximately 10.2% of Australia's population (22,477,000). Using the cost of hospital discharges for the 2010 cohort, estimated as between AUD 173 million (RD-related) and 395 million (all discharges), inpatient hospital discharges for a cohort selected from the whole of Australia could be estimated as being in the range of AUD 1.7 billion -AUD 3.9 billion per year. Using 2010 average monetary exchange rates, this would equate to USD 1.8 to 4.1 billion and EUR 1.4 to 3.1 billion for the year.^21^

Although numerous studies have determined the costs of specific RD in particular settings, there is limited population-based evidence of the collective economic burden of RD. These data support other studies that have shown that genetic diseases result in higher rates of hospitalizations, longer lengths of stay, and greater economic burden than for patients without a genetic disease.^22,23^ Reports of the impact of genetic diseases on different types of hospital utilization such as emergency department presentations, intensive care units, and general hospital wards indicate that the proportion of admissions due to genetic diseases ranges from 2 to 11%.^22,23,24,25,26^

The disease classification with the highest number of people discharged from hospital throughout the whole study period was rare developmental defects during embryogenesis (**Table 1**). Interpretation of this result must take into consideration that nearly one-third of the RD codes used to select the whole cohort were in this category. Diseases classified as rare developmental defects during embryogenesis include chromosomal defects that are complex and severely debilitating and that would contribute to high use of hospital services, particularly pediatric services. Linking data in this study to data from the WA Register of Developmental Anomalies could enable further investigation of the use of pediatric hospital services by young people living with specific RD.

The disease category of rare neoplastic diseases had the second highest number of people discharged from hospital during the study period. Rare neoplastic diseases mirror the challenges that rare conditions collectively present in clinical practice. Notably, these include a lack of knowledge and experience among practitioners and pathologists^27^ leading to late or incorrect diagnosis, difficulties accessing clinical expertise, and appropriate referral pathways and treatments.^28,29,30,31,32^

The whole cohort of this study represents 2.0% of the state population in 2010. This is comparable to a study by Mazzucato et al. that extrapolated prevalence data calculated from a population-based registry to estimate that 1.3 to 2% of the European Union population are living with an RD.^6^ However, this is considerably lower than the usually reported estimates of 6–8% of the population living with an RD.^2,33^ Results from this study should be considered as minimum values for reasons that include the following: (i) the RD resource set used in this study contained only 467 of the estimated 6,000 to 8,000 RD, (ii) RD are likely to be incorrectly or insufficiently coded in administrative data sets because physicians may not know or use appropriate codes for certain RD,^12^ and (iii) people who used outpatient, general practitioner, emergency, and allied health services without inpatient services were not included. Although it is difficult to estimate the extent, these factors could contribute to a significant underestimate of the number of people in WA with an RD.

Substantial elements of the shortfalls of RD coding internationally will be addressed through the next release of ICD codes (ICD-11).^5^ Specifically, Orphanet coding is expected to be formally integrated within ICD-11. This means there will be an internationally accepted and comprehensive data-classification system that supports RD if and when ICD-11 is adopted and implemented. Health-data systems may consider earlier incorporation of Orpha codes into data collections so that as ICD-11 is introduced into health-data collections, there will be the capacity to continue to effectively record and report local RD data. Such actions are consistent with the European Commission Expert Group on RD recommendation adopted in November 2014 for national health-care coding systems to consider using Orpha codes in addition to ICD-10 codes when an RD has no specific ICD-10 code.^5,34^

Several definitions of RD are in use around the world, and many of these refer to both the prevalence and severity of burden—in particular, that RD are life-threatening or chronically debilitating.^35^ Other studies have also reported that determining whether a disease was rare or common was difficult.^36^ The study reported here developed and implemented an RD resource set that was designed to complement the coding used in the specific data collections in WA. Although tailored, this approach may be generalizable to other health systems and populations. The RD resource set used a definition of RD based exclusively on a prevalence of less than 1 in 2,000 people. As a result, the list includes a mixture of both acute and chronic diseases with varying degrees of severity. Furthermore, we were unable to determine when the cohort members were first diagnosed with an RD and whether the condition is lifelong or resolves following treatment. The study makes the assumption that when a person is hospitalized for an RD, they continue to be afflicted with that disease during the study period. Additionally, because of inconsistencies in the way RD codes are coded in hospital records, we were unable to definitively determine which discharges for the cohort were directly related to their RD and which were not. To address this, we examined summary data for all discharges for the cohort as well as the subset of discharges for which an RD code is recorded in a diagnosis field.

This study used linked health data to provide a mechanism to explore the hospital service use of people living with RD. Using a data-linkage infrastructure meant that our study could identify people living with RD and examine their inpatient hospital service use over more than a decade across a statewide health system. Although the methodology has some acknowledged limitations, the findings make an important contribution to the understanding of the collective impact of RD. We have addressed some of the gaps in epidemiological data regarding the collective impact of RD for which evidence is currently limited. This study highlights the marked disparity between the proportion of the population with an RD and their combined cost to the state health system. Along with recent findings that suggest that not all health-care needs of people living with RD are being met,^37^ these data further support the need for improved access to early diagnosis and care coordination for patients with RD. Recognizing RD as a public health priority, the data presented here have been instrumental in guiding clinical service delivery of the Rare and Undiagnosed Diseases Diagnostic Service.^38^ Furthermore, this evidence has informed policymakers in the development of public health strategies, such as the WA Rare Diseases Strategic Framework.^39^

## Disclosure

The authors declare no conflict of interest.

## Figures and Tables

**Table 1:**
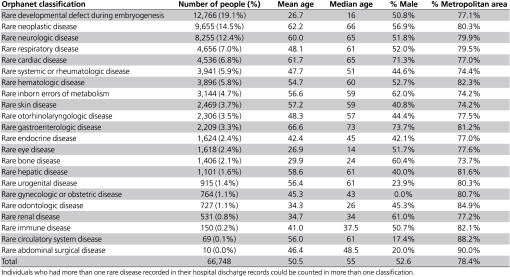
Demographic characteristic of members of the whole cohort by Orphanet classification in decreasing order of frequency

**Table 2:**
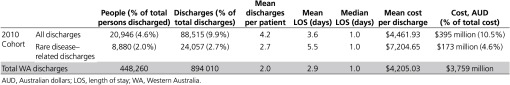
Numbers of people and discharges, LOS and costs for 2010 cohort members and the WA general population during 2010

**Table 3:**
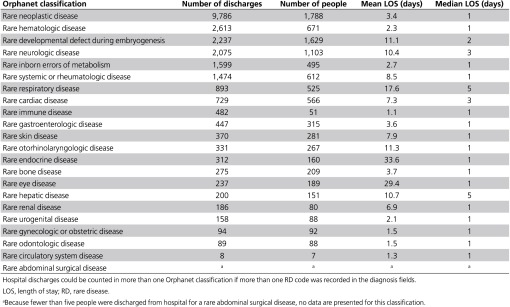
Numbers of RD-related discharges and patients discharged from hospital for an RD-related discharge and LOS of RD-related discharges for 2010 cohort members by Orphanet classification in decreasing order of frequency
